# Accurate GW frontier orbital energies of 134 kilo molecules

**DOI:** 10.1038/s41597-023-02486-4

**Published:** 2023-09-05

**Authors:** Artem Fediai, Patrick Reiser, Jorge Enrique Olivares Peña, Pascal Friederich, Wolfgang Wenzel

**Affiliations:** 1 Institute of Nanotechnology, Karlsruhe Institute of Technology, Hermann-von-Helmholtz-Platz 1, 76344 Eggenstein-Leopoldshafen, Germany; 2 Nanomatch GmbH, Griesbachstraße 5, 76185 Karlsruhe, Germany; 3 Institute of Theoretical Informatics, Karlsruhe Institute of Technology, Am Fasanengarten 5, 76131 Karlsruhe, Germany

**Keywords:** Physical chemistry, Electronic structure of atoms and molecules

## Abstract

HOMO and LUMO energies are critical molecular properties that typically require high accuracy computations for practical applicability. Until now, a comprehensive dataset containing sufficiently accurate HOMO and LUMO energies has been unavailable. In this study, we introduce a new dataset of HOMO/LUMO energies for QM9 compounds, calculated using the GW method. The GW method offers adequate HOMO/LUMO prediction accuracy for diverse applications, exhibiting mean unsigned errors of 100 meV in the GW100 benchmark dataset. This database may serve as a benchmark of HOMO/LUMO prediction, delta-learning, and transfer learning, particularly for larger molecules where GW is the most accurate but still numerically feasible method. We anticipate that this dataset will enable the development of more accurate machine learning models for predicting molecular properties.

## Background & Summary

The availability of a large datasets of sufficiently *accurate* values of frontier orbital energies (i.e., highest occupied and lowest unoccupied orbitals, HOMO and LUMO, respectively) or rather ionization energies (ionization potential and electron affinity, IE and EA, respectively) is a prerequisite for the virtual design of molecules using data-driven, in particular machine learning based, approaches. Virtual materials design is relevant for many applications, ranging from organic electronics^1,2^, functional materials^3^ and thermo-electrics^4^ to homogeneous catalysis^5^.

A ubiquitous method suitable to compute IP and EA in the course of high-throughput screening is density functional theory (DFT)^6^. In DFT, the many-body system of interacting electrons is replaced with a system of non-interacting quasi-particles in the field of the exchange-correlation potential (*V*_*xc*_[*n*]), which is a unique functional of the electron density *n*. Although exact in theory, practical DFT requires severe approximations of *V*_*xc*_[*n*], which can be represented as a chain of progressively more accurate (and more expensive) approximations called Jacob’s ladder^7^. Its first rungs, local density approximation (LDA) and generalized gradient approximation (GGA) are the most widely used approximations. It is well known, however, that these approximations systematically underestimate fundamental HOMO-LUMO gaps by up to 5 eV^8^. Unfortunately, neither the highest implemented rungs of Jacob’s ladder^9^, nor empirical functionals, nor hybrid functionals can closely approach chemical accuracy (1 kcal/mole = 0.0434 eV)^10^.

In contrast to DFT, the GW method allows to systematically increase the accuracy of computing single-particle excitation spectra (including EA and IP) by eliminating some critical problems of DFT, e.g. the interpretation of HOMO and LUMO quasi-particle energies as -IP and -EA, which is an assumption that does not hold in all general cases^11,12^. According to recent reports^13–15^, GW accuracy on various test sets reaches 0.1(0.2) eV, a factor of 2(4) larger than the chemical accuracy.

Here we use the non-self-consistent GW (G_0_W_0_) and eigen-value-self-consistent GW (denoted as GW) based on GGA DFT (namely the PBE exchange-correlation functional^16^) as an initial guess for GW. These two methods are later denoted as G_0_W_0_@PBE and GW@PBE, respectively. A discussion on theoretical details of the GW method can be found in the Supplementary Information. Our data includes HOMO/LUMO and IP/EA energies computed at various levels of theory, ranging from GGA DFT with aug-cc-DZVP basis set to self-consistent GW@PBE extrapolated to the basis set limit. We explain the structure of the dataset, and analyze as well as compare the distribution of energy levels across various levels of theory. Finally, the quality of the basis set limit scheme is analyzed, and results obtained from the quantum chemistry package CP2K^17^ are compared to Gaussian 09 calculations^18^. Notably, this dataset represents the largest collection of GW simulations reported in literature to date. While the accuracy of the method used to compute HOMO/LUMO in original QM9 dataset^19^ is low when compared to experimental results, our reported GW IP/EA energies can be used for machine learning methods that are aimed at accurately predicting ionization energies of small molecules.

## Methods

HOMO and LUMO levels of the whole QM9 dataset molecules were computed in this work using the correlation-consistent basis set aug-cc-DZVP^20^ and the PBE functional^16^ followed by eigenvalue self-consistent GW calculations as implemented in CP2K^21^, which takes the PBE solution as an initial guess (GW@PBE). The same procedure has been repeated for the aug-cc-TZVP basis set. With the GW results from two basis sets we extrapolate the energy to the infinite basis set limit, assuming that the energy is proportional to 1/*N* with *N* being the number of the basis functions^21^. We report HOMO/LUMO energies computed at the level of PBE, G_0_W_0_, GW, each with the two mentioned basis sets together with the corresponding extrapolated values. The notation and dataset labels for HOMO and LUMO orbital energies as computed with DFT as well as GW are summarized in Table 1.

**Table 1:** Notations used for orbital/quasiparticle energies.

Notation in the manuscript	Notation in database files	Meaning	Level of theory
${\varepsilon }_{{\rm{HOMO}}}^{{\rm{DFT}}}$	homo*	HOMO energy computed using PBE functional	Basis sets: aug-cc-DZVP and aug-cc-TZVP *extrapolated* to the basis set limit.
${\varepsilon }_{{\rm{LUMO}}}^{{\rm{DFT}}}$	lumo	LUMO energy computed using PBE functional	Basis sets: the same as above
${\varepsilon }_{{\rm{HOMO}}}^{{\rm{GW}}}$	occ_scf	GW quasiparticle energy of the HOMO computed self-consistently with the PBE starting guess	Basis sets: the same as above GW: quasiparticle eigenvalue-only self-consistent with PBE as an initial guess
${\varepsilon }_{{\rm{LUMO}}}^{{\rm{GW}}}$	vir_scf	GW quasiparticle energy of the LUMO computed self-consistently with the PBE starting guess	Basis sets and GW: as above
${\varepsilon }_{{\rm{HOMO}}}^{{{\rm{G}}}_{0}{{\rm{W}}}_{0}}$	occ_0	G_0_W_0_ quasiparticle energy of the HOMO with the PBE starting guess	Basis sets: the same as above GW: “one-shot” GW with PBE initial guess (not self-consistent).
${\varepsilon }_{{\rm{LUMO}}}^{{{\rm{G}}}_{0}{{\rm{W}}}_{0}}$	vir_0	G_0_W_0_ quasiparticle energy of the LUMO with the PBE starting guess	Basis sets and GW: the same as above
${\widetilde{\varepsilon }}_{{\rm{HOMO}}}^{{{\rm{G}}}_{0}{{\rm{W}}}_{0}}$	occ	G_0_W_0_ quasiparticle energy of the HOMO with the PBE starting guess, assuming the HOMO at PBE remains HOMO at G_0_W_0_ level (not, for instance, HOMO-1)	Basis sets: the same as above GW: “one-shot” GW with PBE initial guess (not self-consistent).
${\widetilde{\varepsilon }}_{{\rm{LUMO}}}^{{{\rm{G}}}_{0}{{\rm{W}}}_{0}}$	vir	G_0_W_0_ quasiparticle energy of the LUMO with the PBE starting guess, assuming the LUMO at PBE remains LUMO at G_0_W_0_ level (not, for instance, HOMO + 1)	Basis sets and GW: as above
${\varepsilon }_{ < {\rm{orbital}} > }^{ < {\rm{method}} > }\left(2\right)$, ${\varepsilon }_{ < {\rm{orbital}} > }^{ < {\rm{method}} > }\left(3\right),$ ${\varepsilon }_{ < {\rm{orbital}} > }^{ < {\rm{method}} > }\left(4\right)$	<name>[2], <name>[3], <name>[4] where <name> is one of the notations from above plus “s” in the end, e.g.: homos[2] is ${\varepsilon }_{{\rm{LUMO}}}^{{\rm{DFT}}}\left(2\right)$	Energies, computed for a specific basis set. Method depends on <orbital> and <method>. Possible values: < orbital>: HOMO or LUMO < method>: DFT or GW	Basis set: (2): aug-cc-DZVP (3): aug-cc-TZVP (4): aug-cc-QZVP

^*^Two extrapolation methods are used to obtain energy levels in the infinite basis set limit. Method 1: ~1/*n*, *n* being the number of basis functions. Method 2: ~1/*N*^3^ with *N* being the basic set size (*i.e*., DZ: 2, TZ: 3, QZ: 4). They are saved as a list, [<method 1>, <method 2>]. Assumptions of method 1 are found to be empirically better, thus it is used throughout the paper.

Although the extrapolation to the basis set limit at the PBE level was performed, it was not actually necessary as the convergence was essentially reached at the level of the aug-cc-DZVP basis set. However, it should be noted that GW HOMO/LUMO energies exhibit slower basis-set convergence^21^, and the extrapolation is essential to attain the nominal GW accuracy.

We employ CP2K Gaussian Augmented Plane Wave (GAPW) method for both DFT and GW simulations. DFT total energies convergence criterion is 10^−6^ Hartree. Realspace grids settings: The cutoff of the finest grid level (CUTOFF) is 500 Ry, the number of multigrids (NGRIDS) is 5; the relative cutoff (REL_CUTOFF) is set to 50 Ry. The simulation cell size (ABC) is set to be 10 Angstroms larger than the linear size of the molecule.

GW simulations were performed using 50 quadrature points (QUADRATURE_POINTS) in resolution-of-identity Random Phase Approximation (RI-RPA) as a default value, crossing search (CROSSING_SEARCH) is set to NEWTON. These simulations converged for about 99% of all molecules (132,151 molecules of 133,885). If the self-consistent quasiparticle solutions were not found within the iteration limit of 20 or the GW algorithm returned NaN values (manifestation of the instability issues) settings were changed: (1) more quadrature points were set: 100, 200, or 500; (2) CROSSING_SEARCH is set to BISECTION instead of NEWTON; (3) if this did not lead to convergence, CUTOFF/REL_CUTOFF was increased to 1000/50, respectively; (4) at last, the Fermi level offset (FERMI_LEVEL_OFFSET) with a default value of 0.02 Hartree set to 0.04 Hartree. As a result, 1351/150/233 molecules converged with 100/200/500 QUADRATURE_POINTS. An example of the default input file for molecule 123456 of the dataset is provided in Supplementary Information. The selection of the numerical settings, as referred to, can be found detailed in Supplementary Table 1. Supplementary Figure 1 further provides a justification for our chosen values of the CUTOFF and REL_CUTOFF parameters.

## Data Records

The dataset is available at Figshare (https://figshare.com/articles/dataset/Accurate_GW_frontier_orbital_energies_of_134_kilo_molecules_of_the_QM9_dataset_/21610077)^22^. The data can be found within the zip archive. Within this archive, the generated data is stored under the filename “db_new_qm9_gw.yaml.” The primary keys in this dictionary correspond to the molecule identifiers, such as “000001,” “000002,” etc., as found in the original QM9 dataset. Each of these primary keys is associated with a dictionary containing the generated data. These secondary dictionaries have keys representing the specific quantities presented, with their corresponding values being the computed results. The meanings and notations of these keys, consistently used throughout this manuscript, are explained in Table 1.

## Technical Validation

### Orbital and quasiparticles energies in the basis set limit

**Fig. 1:**
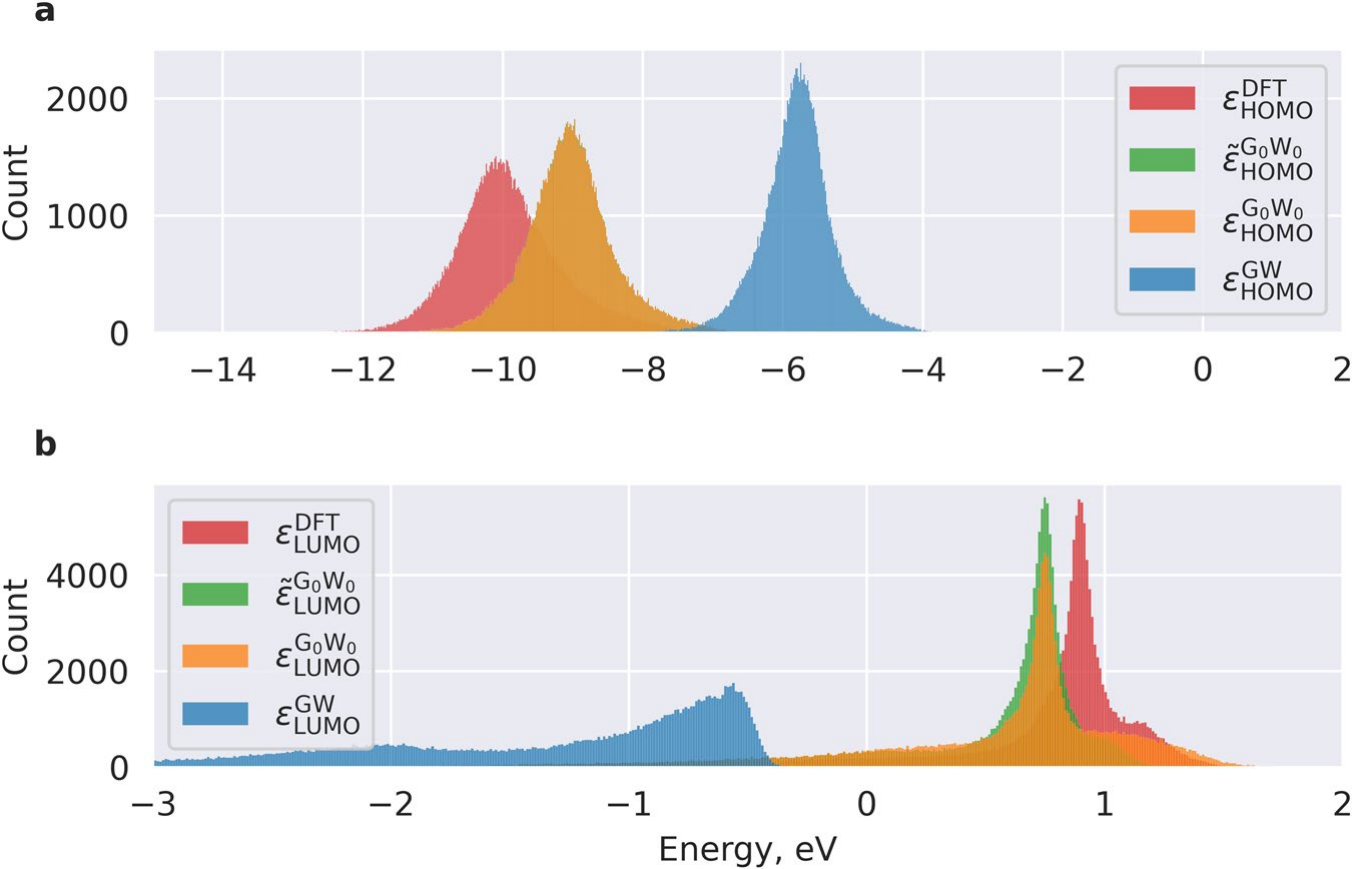
Distribution of frontier orbital energies computed at various levels of theory from DFT to self-consistent GW. (**a**) HOMO. (**b**) LUMO. For notations see Table 1. In (**a**),the green distribution is obscured by the yellow one, as they are almost identical and only slightly differ.

Figure 1 shows the distribution of the PBE and GW HOMO/LUMO energies in the infinite basis set limit. The obtained HOMO position depends on the level of the theory. The systematic difference between PBE and GW level of theory is considerable: DFT with the PBE functional yields a mean HOMO energy of −5.79 eV, while G_0_W_0_@PBE yields a mean HOMO energy of −9.02 eV, which is approximately 3.2 eV lower. GW@PBE is on average approximately 0.9 eV lower than G_0_W_0_@PBE and yields a mean HOMO energy of −9.91 eV. Noticeable is the difference between the distribution of ${\varepsilon }_{{\rm{LUMO}}}^{{{\rm{G}}}_{0}{{\rm{W}}}_{0}}$ and ${\widetilde{\varepsilon }}_{{\rm{LUMO}}}^{{{\rm{G}}}_{0}{{\rm{W}}}_{0}}$ in the energy range between 1 eV and 1.5 eV. This means that many molecules with positive LUMO energy change the order of orbitals. Almost no such effect can be observed for the HOMO energy distributions.

**Fig. 2:**
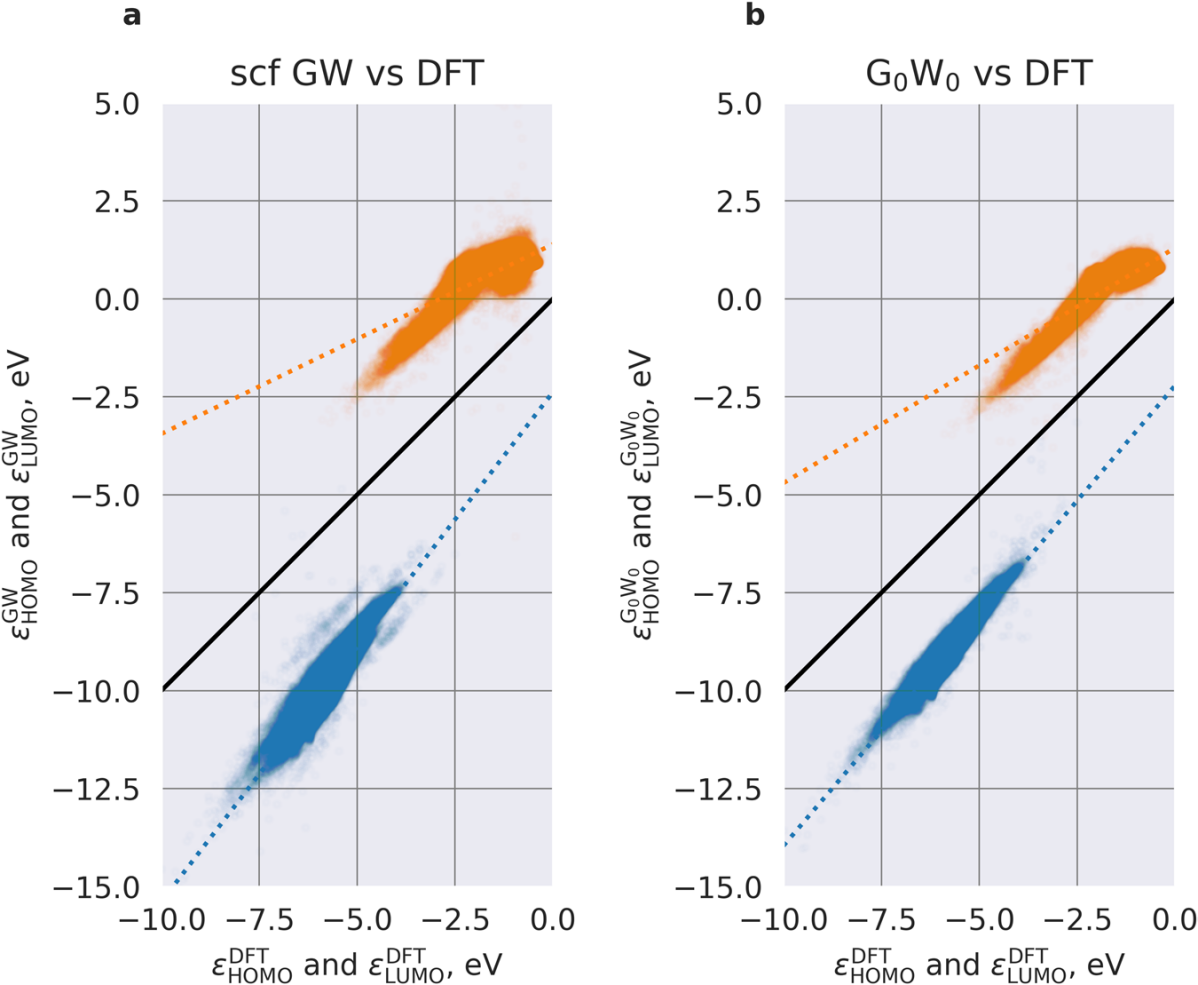
Pair correlation plots of frontier orbitals as computed with GW and DFT methods. (**a**) eigenvalue self-consistent GW vs. DFT. (**b**) “one-shot” GW (G_0_W_0_) vs. DFT.

Figure 2 shows the correlation of GW quasiparticle energies to corresponding DFT orbitals energies. While a few electron-volts difference between DFT and GW methods was obvious from Fig. 1, linear regression fits in Fig. 2 show that the difference between GW and DFT contains large molecule-specific components. For instance, the average difference between ${\varepsilon }_{{\rm{LUMO}}}^{{\rm{GW}}}$ and ${\varepsilon }_{{\rm{LUMO}}}^{{\rm{DFT}}}$ depends on the orbital energy: it increases as ${\varepsilon }_{{\rm{LUMO}}}^{{\rm{DFT}}}$ decreases (the slope of the dotted regression line in Fig. 2 is 0.48). Additionally, there is a large spread of the data (the mean absolute deviation of ${\varepsilon }_{{\rm{LUMO}}}^{{\rm{GW}}}$ distribution is 0.34 eV). DFT HOMO energies correlate better to GW HOMOs than LUMO levels, e.g. for HOMOs, the coefficients of determination *R*^2^ are 0.79 and 0.90 for GW and G_0_W_0_, whereas for LUMOs *R*^2^ are 0.61 and 0.77 for GW and G_0_W_0_, respectively. This linear regression analysis reveals that there is no straightforward correlation between the HOMO energy computed at the GGA and GW levels. The correlation for LUMO is even weaker, likely because predicting LUMO is more challenging than HOMO, given its increased sensitivity to approximations, delocalization, screening effects, and chemical diversity (LUMO variability is generally larger in the same chemical space than HOMO).

### Benchmarking and choosing basis set limit extrapolation schemes

Due to the slow basis set convergence of quasiparticle HOMO and LUMO energies in GW calculations, extrapolation to the complete basis set limits was carried out. GW energies of all QM9 molecules were computed using two all-electron basis sets of a different size: aug-cc-DZVP and aug-cc-TZVP, and then extrapolated using two basis set extrapolation schemes^13^. *Scheme 1* employs a linear fit on the HOMO or LUMO values versus the inverse cardinal number of the basis set *N*_basis_ (GW HOMO/LUMO energy is assumed to be proportional to 1/*N*_basis_). *Scheme 2* extrapolates HOMO/LUMO energies against 1/*N*_card_^3^ where *N*_card_ is the cardinal number of the basis set (for example 2 for aug-cc-DZVP, 3 for aug-cc-QZVP, etc.).

To test the quality of the extrapolation from these two relatively smaller aug-cc basis sets, one hundred pseudo-random molecules from the QM9 dataset were simulated with the larger aug-cc-QZVP basis set.

The extrapolated GW HOMO and LUMO energies analyzed in this paper is based on *Scheme* 1, although the data set contains extrapolated values for both *Scheme* 1 and *Scheme* 2. For Scheme 1, the smallest mean absolute error (mae) is reached for ${\varepsilon }_{{\rm{HOMO}}}^{{{\rm{G}}}_{0}{{\rm{W}}}_{0}}$ of 6.0 meV, more than an order of magnitude more than the GW method accuracy. The worst extrapolation quality is observed for ${\varepsilon }_{{\rm{LUMO}}}^{{\rm{GW}}}$ with a mae of 37.0 meV. However, this is still acceptable, as it is a few times smaller than the GW mean error (around 100…200 meV^13^). The extrapolation errors are defined as the normalized sum of the absolute differences of the extrapolated values computed with the use of two (aug-cc-DZVP, aug-cc-TZVP) and three (aug-cc-DZVP, aug-cc-TZVP, and aug-cc-QZVP) basis sets:

$${{\rm{mae}}}_{ < {\rm{orbital}} > }^{ < {\rm{method}} > }=\frac{1}{{N}_{{\rm{mol}}}}\sum _{i}\left|{{\rm{\varepsilon }}}_{ < {\rm{orbital}} > ,i}^{ < {\rm{method}} > }\left(2,3,4\right)-{{\rm{\varepsilon }}}_{ < {\rm{orbital}} > ,i}^{ < {\rm{method}} > }\left(2,3\right)\right|$$

where <method> is either GW or G_0_W_0_, <orbital> is either HOMO or LUMO, *i* is the molecular index, *N*_mol_ is the number of molecules, which is 100. ${{\rm{\varepsilon }}}_{ < {\rm{orbital}} > ,i}^{ < {\rm{method}} > }\left(2,3,4\right)$ and ${{\rm{\varepsilon }}}_{ < {\rm{orbital}} > ,i}^{ < {\rm{method}} > }\left(2,3\right)$ denote extrapolated energies computed using three and two basis sets, respectively. ${{\rm{\varepsilon }}}_{ < {\rm{orbital}} > ,i}^{ < {\rm{method}} > }\left(2,3\right)$ is identical to ${{\rm{\varepsilon }}}_{ < {\rm{orbital}} > ,i}^{ < {\rm{method}} > }$, and is added here for clarity.

**Fig. 3:**
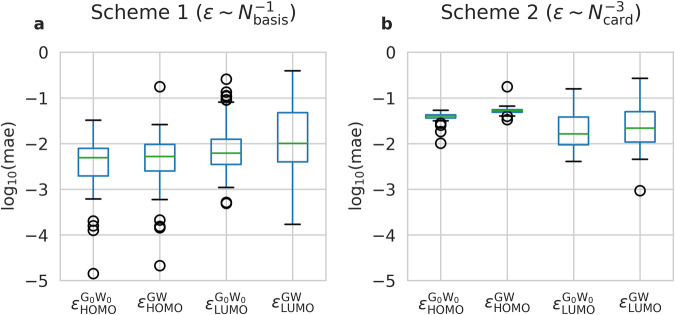
Visualization of the extrapolation errors of HOMO/LUMO computed at GW@PBE and G0W0@PBE levels. (**a**). Scheme 1. (**b**). Scheme 2. The extrapolation errors are computed for 100 random molecules from the QM9 dataset. They are defined as the normalized sum of the absolute differences of the extrapolated values computed with the use of two (aug-cc-DZVP, aug-cc-TZVP) and three (aug-cc-DZVP, aug-cc-TZVP, and aug-cc-QZVP) basis sets. Scheme 1 is up to one order of magnitude more accurate than Scheme 2. In the box plots, the box represents the interquartile range (IQR), containing data between the 25% and 75% percentiles, with the median indicated by a line inside the box. Whiskers extend from the box to the minimum and maximum values within 1.5 times the IQR, and outliers beyond the whiskers are displayed individually.

Unfortunately, the overall acceptable mean absolute error magnitude is accompanied with a few outliers (see Fig. 3), which are much more pronounced for LUMO than HOMO extrapolation errors. The outliers are observed for the unbounded states (positive LUMO values), as depicted in Supplementary Figure 2.

### Benchmark calculations using B3LYP

**Fig. 4:**
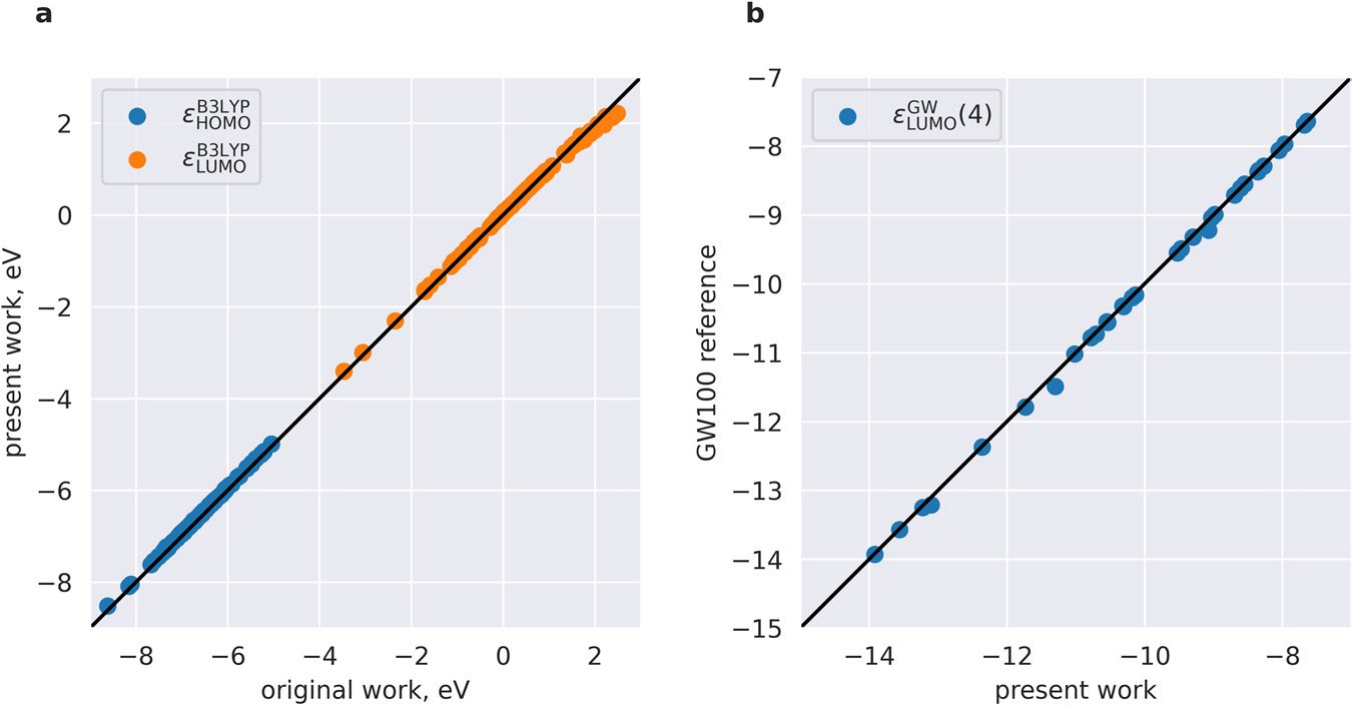
Benchmarking calculations. (**a**) Correlation plot of HOMO and LUMO, contained in the original dataset (Gaussian 09) and here (CP2K). Theory level: B3LYP/6-31 G(2df,p). (**b**) Correlation plot of GW@PBE HOMOs, as deposited in the GW100 data set^23^ in comparison with the present work (CP2K). Theory level: Self-consistent GW@PBE with a def2-QZVP basis set.

Original simulations of HOMO and LUMO energies in the QM9 data set were performed using the B3LYP functional and a 6–31 G(2df,p) basis set using the Gaussian 09 software [Frisch, M. J. *et al. Gaussian 09, Revision d.01* (Gaussian, Inc., 2009).]^18^. In addition to the aforementioned computational protocol for DFT/GW simulations, we also performed B3LYP/6-31 G(2df,p) calculations to estimate differences between CP2K^21^ used here and the original work (Gaussian 09). Results are shown in Fig. 4a for 100 randomly selected molecules from the QM9 dataset. While perfect correlation is observed for HOMOs (mean value of the absolute HOMO differences is 11 meV), LUMO values demonstrate worse correlation (mean value of the absolute LUMO differences is 70 eV). For LUMOs which have energies exceeding 1 eV, the orbital energies computed in this work are systematically lower than the original QM9 energy, which could be due to the fact that CP2K uses mixed localized/plane-wave basis sets to represent electron density, which is different in Gaussian.

### Benchmark calculations for GW100 dataset

The *GW*100 ^13^ dataset is a dataset of small molecules used to benchmark GW implementation in various quantum chemistry codes. The GitHub repository^23^ contains, among others, HOMO quasiparticles energies computed using CP2K self-consistently at GW@PBE level using def2-QZVP basis set^24^. Figure 4b compares the organic molecules within GW100 with CP2K simulations at the same theory level. However, the exact equivalence of all computational settings cannot be assured as the full CP2K input files are not available. Apart from the outlier molecule Carbon tetrafluoride, named 75-73-0 in GW100 data repository (for which the error is 71 meV), the observed differences are small, with a mean unsigned error of 28 meV (including the outlier), which is substantially smaller than the accuracy of the GW method itself.

### Computational resources and scaling

**Fig. 5:**
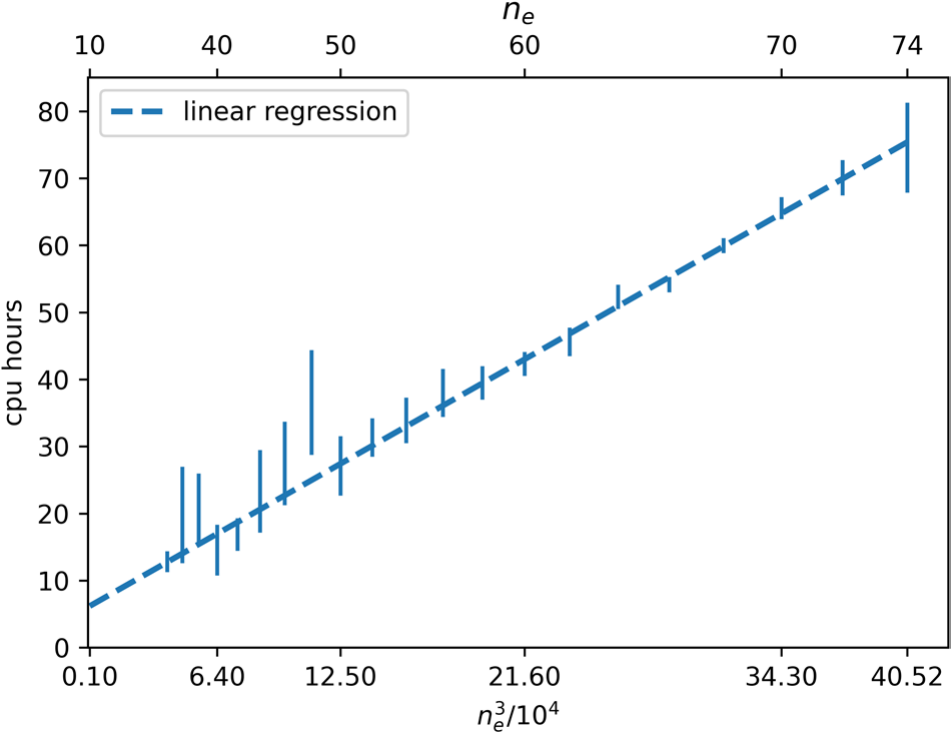
Scaling of the computation time (*cpu hours*) depending on the cubic number of electrons in a molecule, *n*_*e*_^3^/10^4^_._ The upper horizontal axis is nonlinear, and represents the number of electrons *n*_*e*_. Cubic cpu time scaling (O(*N*^3^)) is observed for GW implementations.

Overall, it took 7,439,925 cpu hours to perform DFT and GW simulations in order to generate the scientific data reported. The total cpu time to make DFT and GW simulations for one molecule scales as *n*_*e*_^3^ with *n*_*e*_ being the number of electrons of the molecule (see Fig. 5). More details are visualized in Supplementary Figure 3, including distribution of computational time splitted by the different cpu model specifications. Hardware specifications used in this work are listed in Supplementary Table 2.

## Usage Notes

We presented accurate values of HOMO and LUMO of 134 kilo molecules, computed with an eigenvalue self-consistent GW method in a basis set limit, along with auxiliary data: G_0_W_0_, and DFT values of HOMO and LUMO orbitals. This data can be used to benchmark machine-learning methods, which aim at the accurate prediction of single-particle excitation energies. It contains many more molecules than the standard GW100 data set, and thus can also be used to benchmark new and existing GW codes.

### Supplementary information

Supplementary Information

## Data Availability

An input file for the CP2K calculations can be found in the Supplementary Information. Further code is not required to reproduce the data presented in this article.
