# Supplementary material for: Accurate GW frontier orbital energies of 134 kilo molecules
Source: Sci Data. 2023 Sep 5;10:581. doi: 10.1038/s41597-023-02486-4 (PMC10480222; doi:10.1038/s41597-023-02486-4)
Supplement: Supplementary file 1 — Supplementary Information [file 41597_2023_2486_MOESM1_ESM.pdf]

Supporting Information

GW frontier orbital energies of 134 kilo molecules

Artem Fediai, Patrick Reiser, Jorge Enrique Olivares Peña, Pascal Friederich, Wolfgang Wenzel

Note 1. Short theory review of GW

The GW method[1–3] provides an approximation to the real many-body excitation spectra using single quasiparticle Green's functions G[4]. The effects of exchange (Pauli repulsion) and correlation (screening) are taken into account through an energy dependent self-energy $\mathbf{\Sigma}_{GW}(E) = \mathbf{\Sigma}_x + \mathbf{\Sigma}_c(E)$ that depends on the Green's function. The Green's function enters a Dyson type of equation connecting the non-interacting (Hartree type) Green's function $G_H(z)$ with the full interacting Green's function: $G(z) = G_H(z) + G_H(z)\mathbf{\Sigma}_{GW}(z)G(z),$ where z is a complex number. In the GW approximation, the vertex corrections as defined by Hedin[1] are ignored and therefore one can calculate the self-energy using the second Hedin's equation with a dynamically screened Coulomb potential W which we write symbolically as : $\Sigma = iGW$. The GW approximation can be viewed as the generalization of the Hartree-Fock method where exchange energy is represented as $V_x = iGV$ with V_x being a bare interaction potential. Bare and screened potentials differ by dielectric function ϵ: $W = \epsilon^{-1}V$. The self-energy includes the effects of the static and dynamic screening (correlations) missing in the Hartree-Fock method and "underrepresented" in the practical DFT implementations.

Practical solution for the quasiparticles energy levels in GW is a self-consistent problem (thus another name for this GW implementation is scGW), just like the solution of the Kohn-Sham equations. This self-consistent solution (solving Hedin's equations with an initial guess on the Green's function) does not depend on the initial choice of the reference system which can be taken from DFT or Hartree-Fock methods[5]. The problem is that the "full" solution is computationally demanding and the computation cost of a canonical GW growth as $O(N^6)$ with the number of particles. In practice, the so-called "full" self-consistent GW is rarely used. A "one-shot" GW, called G_0W_0 , is the least expensive scheme, where the self-energy $\mathbf{\Sigma}$ is calculated once using the Green's function obtained with the initial guess (DFT in many cases) G_{DFT} , in other words, just one iteration of the scGW is performed. In contrast to the full GW method, G_0W_0 does depend on the initial guess of the reference system, thus, excellent DFT convergence is a must to obtain reliable results. There exist other flavors of GW which are sometimes called "partial self-consistent"[5] approaches like the so called quasiparticle-self-consistent GW (qsGW) and the quasiparticle eigenvalue-only self consistent GW (evGW). Although one expects the accuracy of the self-consistent methods to be be higher than that of G_0W_0, G_0W_0 may be fairly accurate if using a reasonable initial guess, and outperforms scGW (in terms of memory and computational time resources) when screening a large amount of molecules. qs-GW and ev-GW lead to very similar results.

Note 2. Example of cp2k input file

```
&GLOBAL
  EXTENDED_FFT_LENGTHS TRUE
  PRINT_LEVEL LOW
  PROJECT_NAME 2
  RUN_TYPE ENERGY
&END GLOBAL
&FORCE_EVAL
  METHOD QUICKSTEP
  &DFT
        POTENTIAL_FILE_NAME POTENTIAL
        UKS FALSE
        MULTIPLICITY 1
        CHARGE 0
        BASIS_SET_FILE_NAME BASIS_CC_AUG_RI_NEW
        &SCF
        MAX_SCF 100
        EPS_SCF 1e-06
        SCF_GUESS RESTART
        ADDED_MOS 1000
        &DIAGONALIZATION T
        &END DIAGONALIZATION
        &MIXING
        METHOD BROYDEN_MIXING
        ALPHA 0.2
        BETA 1.5
        NBUFFER 8
        &END MIXING
        &END SCF
        &QS
        EPS_DEFAULT 1e-10
        EPS_PGF_ORB 1e-05
        METHOD GAPW
        &END QS
        &MGRID
        NGRIDS 5
        CUTOFF 500
        REL_CUTOFF 50
        &END MGRID
        &XC
        &XC_FUNCTIONAL PBE
        &END XC_FUNCTIONAL
        &WF_CORRELATION
        METHOD RI_RPA_GPW
        MEMORY 4000
        GROUP_SIZE 1
        ERI_METHOD OS
        &RI_RPA
        QUADRATURE_POINTS 50
        SIZE_FREQ_INTEG_GROUP -1
        RI_G0W0 TRUE
        &RI_G0W0
               CORR_MOS_OCC 20
               CORR_MOS_VIRT 20
               CROSSING_SEARCH NEWTON
               EV_SC_ITER 20
               RI_SIGMA_X .TRUE.
               ANALYTIC_CONTINUATION PADE
        &END RI_G0W0
```

```
      &HF
              FRACTION 1.0
              &SCREENING
              EPS_SCHWARZ 1e-11
              SCREEN_ON_INITIAL_P FALSE
              &END SCREENING
              &MEMORY
              MAX_MEMORY 500
              &END MEMORY
      &END HF
      &END RI_RPA
      &END WF_CORRELATION
      &END XC
      &POISSON
      POISSON_SOLVER MT
      PERIODIC NONE
      &END POISSON
      &PRINT
      &MO_CUBES
      FILENAME =HOMO.txt
      WRITE_CUBE FALSE
      NLUMO 10
      NHOMO 10
      &END MO_CUBES
      &END PRINT
&END DFT
&SUBSYS
      &CELL
      ABC 16.31807877 15.17847716 14.0643624
      PERIODIC NONE
      &END CELL
      &TOPOLOGY
      COORD_FILE_NAME dsgdb9nsd_123456.xyz
      COORD_FILE_FORMAT xyz
      &CENTER_COORDINATES
      &END CENTER_COORDINATES
      &END TOPOLOGY
      &KIND H
      RI_AUX_BASIS_SET aug-cc-pVDZ-RIFIT
      ELEMENT H
      POTENTIAL ALL
      BASIS_SET aug-cc-pVDZ
      &END KIND
      &KIND C
      RI_AUX_BASIS_SET aug-cc-pVDZ-RIFIT
      ELEMENT C
      POTENTIAL ALL
      BASIS_SET aug-cc-pVDZ
      &END KIND
      &KIND N
      RI_AUX_BASIS_SET aug-cc-pVDZ-RIFIT
      ELEMENT N
      POTENTIAL ALL
      BASIS_SET aug-cc-pVDZ
      &END KIND
      &KIND O
      RI_AUX_BASIS_SET aug-cc-pVDZ-RIFIT
      ELEMENT O
      POTENTIAL ALL
      BASIS_SET aug-cc-pVDZ
      &END KIND
      &KIND F
```

```
        RI_AUX_BASIS_SET aug-cc-pVDZ-RIFIT
        ELEMENT F
        POTENTIAL ALL
        BASIS_SET aug-cc-pVDZ
        &END KIND
        &KIND P
        RI_AUX_BASIS_SET aug-cc-pVDZ-RIFIT
        ELEMENT P
        POTENTIAL ALL
        BASIS_SET aug-cc-pVDZ
        &END KIND
        &KIND S
        RI_AUX_BASIS_SET aug-cc-pVDZ-RIFIT
        ELEMENT S
        POTENTIAL ALL
        BASIS_SET aug-cc-pVDZ
        &END KIND
        &KIND Cl
        RI_AUX_BASIS_SET aug-cc-pVDZ-RIFIT
        ELEMENT Cl
        POTENTIAL ALL
        BASIS_SET aug-cc-pVDZ
        &END KIND
        &KIND Br
        RI_AUX_BASIS_SET aug-cc-pVDZ-RIFIT
        ELEMENT Br
        POTENTIAL ALL
        BASIS_SET aug-cc-pVDZ
        &END KIND
        &KIND B
        RI_AUX_BASIS_SET aug-cc-pVDZ-RIFIT
        ELEMENT B
        POTENTIAL ALL
        BASIS_SET aug-cc-pVDZ
        &END KIND
        &KIND I
        RI_AUX_BASIS_SET aug-cc-pVDZ-RIFIT
        ELEMENT I
        POTENTIAL ALL
        BASIS_SET aug-cc-pVDZ
        &END KIND
  &END SUBSYS
&END FORCE_EVAL
```

Table 1. Choosing convergence parameters for cp2k implementation of GW

parameter	this work	[P. 2016]	[P. 2017]	Reason
Cutoff	500	800	800	GAPW does not require as fine grid as GPW. 300 is what would be sufficient.
rel_cutoff	50	60	800	The same as for using smaller cutoff
Eps_scwartz	1e-11	1e-300	1e-200	TABLE S3. [2017] for 6-anthene (114 atoms) shows that 1 meV accuracy in HOMO-LUMO gap is observed here.
QUADRATURE_ POINTS	50	100	12	Compromise between the accuracy and CPU time (CPU time scales linearly with this number)

References are two manuscript on cp2k implementation of GW method in year 2016 and 2017:

[P. 2016]: Reference[6]

[P. 2017]: Reference[7]

Table 2. Hardware specifications

	CPU Model	Cluster
1	Xeon(R) Gold 6252 CPU @ 2.10GHz	JUSTUS 2
2	Intel(R) Xeon(R) CPU E5-2630 v3 @ 2.40GHz	MLS&WISO
3	Intel(R) Xeon(R) CPU E5-2640 v3 @ 2.60GHz	MLS&WISO
4	Intel(R) Xeon(R) CPU E5-4620 v3 @ 2.00GHz	MLS&WISO
5	AMD EPYC 7702 64-Core Processor	int-nano
6	AMD EPYC 7551P 32-Core Processor	int-nano

Effect of the real space grid on the GW energies

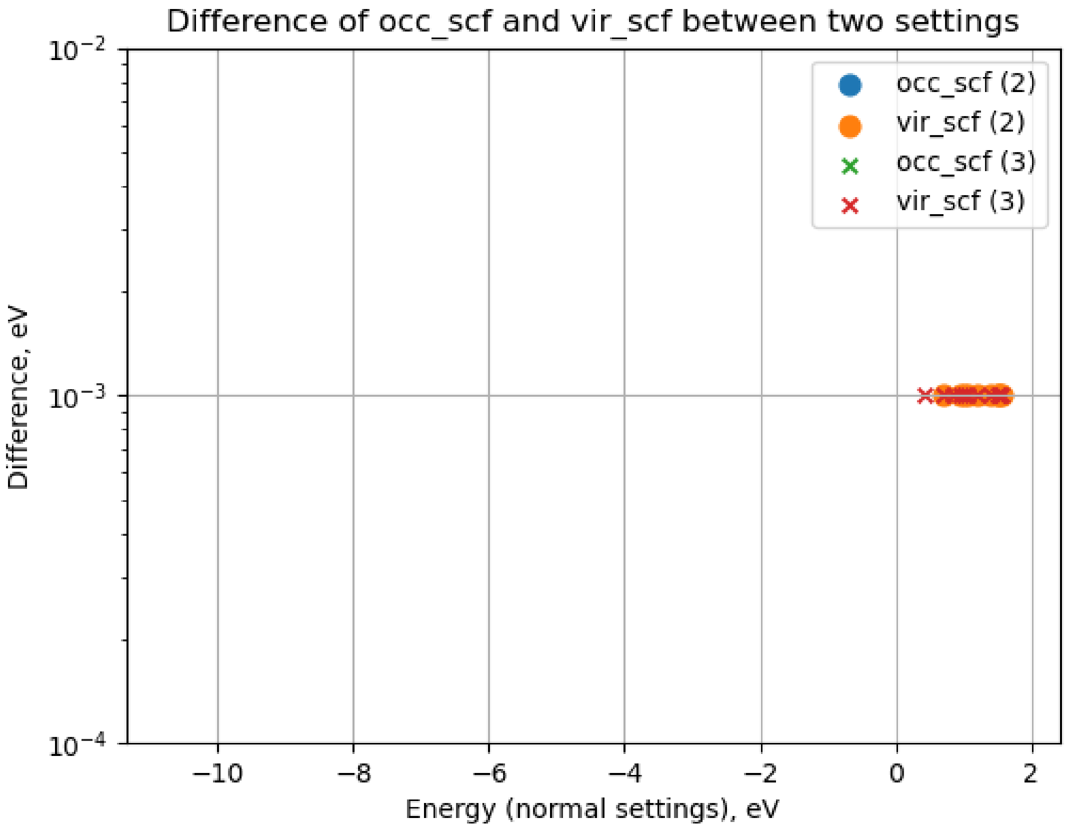

Figure 1. The absolute differences in the computed energies $\epsilon^{\mathrm{GW}}_{\mathrm{HOMO}}(i)$ and $\epsilon^{\mathrm{GW}}_{\mathrm{LUMO}}(i)$ between the "normal" grid settings (CUTOFF=50, REL_CUTOFF=500) used in the manuscript, and a finer grid size setting (CUTOFF=800, REL_CUTOFF=60). Computations are made for 100 random molecules. These are plotted versus the energy of the corresponding orbitals. *i* is the cardinal number of the basis set. The GW orbital energies are output in eV with three decimal places (accuracy 1 meV). Therefore, for all molecules but shown at the plot, the accuracy is higher than 1 meV. For those shown here, the error is exactly 1 meV, which justify using the grid size CUTOFF=500; REL_CUTOFF=50 in the manuscript. Note that an error as large as 1 meV is exclusively observed for virtual unbonded orbitals. When utilizing the aug-cc-DZVP basis set (with cardinal number 2), this non-zero error emerges in 12 molecules out of 100. In contrast, using the aug-cc-TZVP basis set (with cardinal number 3) results in a non-zero error in 10 molecules.

Basis set dependence of the predicted LUMO

It is widely acknowledged that unbounded states (positive LUMO) require an adequate number of diffuse functions for accurate representation. **Figure 2** illustrates the relationship between the extrapolated LUMO magnitude and the standard error of its extrapolation, indicating that the computed values of unbounded LUMO warrant greater caution. Nevertheless, the extrapolation remains acceptable overall.

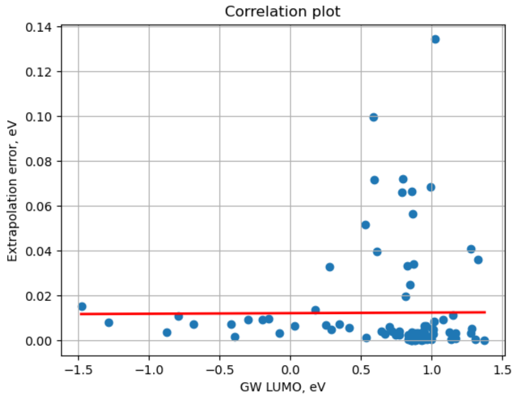

Figure 2. Correlation between extrapolation error and LUMO magnitude for 100 random molecules. The graph demonstrates that the average extrapolation error remains largely unaffected by the LUMO magnitude, even when comparing unbounded (positive) LUMO values to bounded (negative) ones, as evidenced by the red linear regression line. It is important to note that the distribution of positive LUMO values features several outliers, suggesting reduced approximation accuracy when employing aug-cc-DZVP and aug-cc-TZVP basis sets for extrapolating unbounded LUMO values.

CPU time scaling

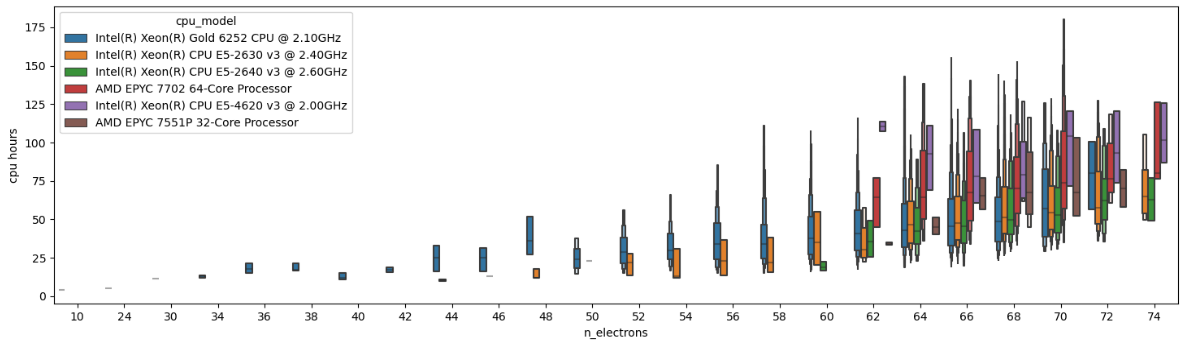

Figure 3. Computational time depending on the number of electrons split by different CPU model specifications. Total amount of computational resources in cpu hours: 7,439,925. This includes computing DFT electron density, which is used as a starting point for GW calculations, and GW calculations themselves. Both are done for two basis sets.

References

1. Hedin, L. New Method for Calculating the One-Particle Green's Function with Application to the Electron-Gas Problem. *Phys. Rev.* **139**, A796–A823 (1965).
2. Aulbur, W. G., Jönsson, L. & Wilkins, J. W. Quasiparticle Calculations in Solids. in *Solid State Physics* (eds. Ehrenreich, H. & Spaepen, F.) vol. 54 1–218 (Academic Press, 2000).
3. Aryasetiawan, F. & Gunnarsson, O. The GW method. *Rep. Prog. Phys.* **61**, 237–312 (1998).
4. Hüser, F., Olsen, T. & Thygesen, K. S. Quasiparticle GW calculations for solids, molecules, and two-dimensional materials. *Phys. Rev. B* **87**, 235132 (2013).
5. van Setten, M. J., Weigend, F. & Evers, F. The GW-Method for Quantum Chemistry Applications: Theory and Implementation. *J. Chem. Theory Comput.* **9**, 232–246 (2013).
6. Wilhelm, J., Del Ben, M. & Hutter, J. GW in the Gaussian and Plane Waves Scheme with Application to Linear Acenes. *J. Chem. Theory Comput.* **12**, 3623–3635 (2016).
7. Wilhelm, J., Golze, D., Talirz, L., Hutter, J. & Pignedoli, C. A. Toward *GW* Calculations on Thousands of Atoms. *J. Phys. Chem. Lett.* **9**, 306–312 (2018).
